# Efficacy of the Mindfulness Meditation Mobile App “Calm” to Reduce Stress Among College Students: Randomized Controlled Trial

**DOI:** 10.2196/14273

**Published:** 2019-06-25

**Authors:** Jennifer Huberty, Jeni Green, Christine Glissmann, Linda Larkey, Megan Puzia, Chong Lee

**Affiliations:** 1 College of Health Solutions Arizona State University Phoenix, AZ United States; 2 Department of Student Affairs University of Redlands Redlands, CA United States; 3 College of Nursing and Health Innovation Arizona State University Phoenix, AZ United States; 4 Behavioral Research and Analytics, LLC Salt Lake City, UT United States

**Keywords:** meditation, mental health, mindfulness, smartphone, technology, mobile phone

## Abstract

**Background:**

College students experience high levels of stress. Mindfulness meditation delivered via a mobile app may be an appealing, efficacious way to reduce stress in college students.

**Objective:**

We aimed to test the initial efficacy and sustained effects of an 8-week mindfulness meditation mobile app—Calm—compared to a wait-list control on stress, mindfulness, and self-compassion in college students with elevated stress. We also explored the intervention’s effect on health behaviors (ie, sleep disturbance, alcohol consumption [binge drinking], physical activity, and healthy eating [fruit and vegetable consumption]) and the feasibility and acceptability of the app.

**Methods:**

This study was a randomized, wait-list, control trial with assessments at baseline, postintervention (8 weeks), and at follow-up (12 weeks). Participants were eligible if they were current full-time undergraduate students and (1) at least 18 years of age, (2) scored ≥14 points on the Perceived Stress Scale, (3) owned a smartphone, (4) were willing to download the Calm app, (5) were willing to be randomized, and (7) were able to read and understand English. Participants were asked to meditate using Calm at least 10 minutes per day. A *P* value ≤.05 was considered statistically significant.

**Results:**

A total of 88 participants were included in the analysis. The mean age (SD) was 20.41 (2.31) years for the intervention group and 21.85 (6.3) years for the control group. There were significant differences in all outcomes (stress, mindfulness, and self-compassion) between the intervention and control groups after adjustment for covariates postintervention (all *P*<.04). These effects persisted at follow-up (all *P*<.03), except for the nonreacting subscale of mindfulness (*P*=.08). There was a significant interaction between group and time factors in perceived stress (*P*=.002), mindfulness (P<.001), and self-compassion (*P*<.001). Bonferroni posthoc tests showed significant within-group mean differences for perceived stress in the intervention group (*P*<.001), while there were no significant within-group mean differences in the control group (all *P*>.19). Similar results were found for mindfulness and self-compassion. Effect sizes ranged from moderate (0.59) to large (1.24) across all outcomes. A significant group×time interaction in models of sleep disturbance was found, but no significant effects were found for other health behaviors. The majority of students in the intervention group reported that Calm was helpful to reduce stress and stated they would use Calm in the future. The majority were satisfied using Calm and likely to recommend it to other college students. The intervention group participated in meditation for an average of 38 minutes/week during the intervention and 20 minutes/week during follow-up.

**Conclusions:**

Calm is an effective modality to deliver mindfulness meditation in order to reduce stress and improve mindfulness and self-compassion in stressed college students. Our findings provide important information that can be applied to the design of future studies or mental health resources in university programs.

**Trial Registration:**

ClinicalTrials.gov NCT03891810; https://clinicaltrials.gov/ct2/show/NCT03891810

## Introduction

Elevated stress has been reported in more than 75% of college students (ie, aged 18-33 years) [1-3], and college students often report higher levels of stress than people of other age groups [4,5]. Some studies showed that more than 85% of college students reported feeling overwhelmed [4,5]. This is likely due to the unique set of stressors and demands they experience as they gain autonomy from their parents (ie, move away leaving family and friends), learn to manage finances, balance an increased academic workload and extracurricular activities, and make career choices [5-7]. These demands may lead to increased anxiety, loneliness, depression, sleep disturbance, and even suicidal ideation [8-10].

Stress is associated with a greater likelihood of suicide attempts [11], which is the second leading cause of death among teens and young adults (aged 15-24 years) [12]. One report found that 60.8% of college students felt overwhelming anxiety, 38.2% felt so depressed that it was difficult to function, and 10.4% seriously considered suicide [13]. Studies suggest that untreated mental health is highly prevalent among college students. One study reported that <25% of college students received treatment for any type of mental health disorder (eg, depression or anxiety) within the past year [14]. Another study reported that only 36% of students with mental health issues had sought treatment [15]. Many students do not seek treatment partly due to the lack of time, privacy concerns, stigma [16], lack of emotional openness [17], and financial constraints [18-21].

Beyond the potential negative impact that elevated stress may have on mental health, stress may also have a negative impact on the physical health of college students and the health behavior they choose during this time [22]. College students with elevated stress may be more likely to experience poor sleep, increased alcohol consumption, less exercise, and unhealthy eating habits [23-25]. There is an urgent need for effective stress management strategies to address the adverse outcomes of stress and the potential barriers to treatment in college students.

Implementing stress reduction programs on college campuses has become a priority [26,27]. Mindfulness-based interventions, in particular, have become more popular on college campuses recently [28] and may be an effective strategy to reduce stress in college students [29]. Mindfulness is defined as the state of being attentive to and aware of what is taking place in the present moment without judgement [30]. Two of the most popular mindfulness-based interventions are mindfulness-based stress reduction (MBSR) and mindfulness-based cognitive therapy (MBCT) [31,32]. Although effective for stress management, these programs may be rigorous, time consuming, and costly, which may not be ideal for college students.

Mindfulness meditation, a component of MBSR and MBCT, alone has shown to have positive benefits—from reducing stress and anxiety to improving overall well-being [29,33]. The mechanisms of mindfulness meditation are unclear but constructs such as increasing self-compassion and mindfulness have been shown to mediate the effect of meditation programs on stress in a variety of populations [34-36]. Meta-analyses have suggested that self-compassion is strongly related to mental health [37] and well-being [38]. However, the stress-reducing effects in college students is less clear. More research is needed to understand what aspects of mindfulness meditation interventions may be working to reduce stress in college students.

Mindfulness meditation may be a practical approach to reduce stress in college students. In a recent narrative synthesis of studies on the effects of mindfulness meditation on stress in college students, 16 of the 22 studies reported significant decreases in self-reported stress [29]. Significant findings were reported in interventions ranging from 2.25 hours to as much as 30 hours of total required participation in meditation over the course of the intervention (eg, 3-8 weeks) [39-44]. Although these findings are promising, several limitations must be noted, including low adherence rates, lack of control groups, and small sample sizes [29]. More studies are needed to address the existing limitations of mindfulness meditation interventions and explore more convenient ways to deliver mindfulness meditation in order to encourage participation and increase accessibility among college students.

Approximately 98% of college-aged students use the internet [45] and 85% own a smartphone [46]. Many college students have expressed being receptive to online mental health treatments, with one study reporting that college students were more likely to seek help online than face-to-face [47]. The development of mobile health apps has been increasing exponentially, and the use of apps has been reported to improve the efficiency of health care delivery and the effectiveness of treatment [48]. It is also important to note that while apps may offer the ability for increased access to health information, remote care, and user autonomy [49], there is a need to ensure safety of users, as there is no regulatory oversight. App users should consider the quality and accuracy of evidence-based information, qualifications of content developers, medical/health claims [50], information sharing or data security [51], informed consent, and user competence (eg, training required to use techniques) [49]. Independent testing of mobile apps is highly warranted.

Mindfulness meditation mobile apps may be a promising approach to reduce stress and address the barriers for stress management reported by college students. A recent study conducted a search for “mindfulness” mobile apps in Apple iTunes and Google Play Store and found a total of 560 apps that were accessible and in English [52]. The top mindfulness apps listed in Apple iTunes (in a current search as of April 2019) was Calm (named “2017 app of the year” by Apple, #2 Health & Fitness app, 4.8/5 star reviews), followed by Aura (#6 Health & Fitness app, 4.7/5 star reviews) and Headspace (#9 Health & Fitness app, 4.9/5 star reviews). Similar results were found in the Google Play Store, with the top mindfulness apps being Headspace (#6 Health & Fitness app, 4.5/5 star reviews), followed by Calm (#8 Health & Fitness app, 4.5/5 star reviews) and Deep Calm (#44 Health & Fitness app, 4.5/5 star reviews). Although the popularity of mindfulness apps is increasing, only a few studies have examined the feasibility and efficacy of such mindfulness-based mobile apps for reducing stress among college students.

The purpose of this study was to test the efficacy [53] of an 8-week mindfulness meditation intervention delivered via a consumer-based mobile app (ie, Calm) as compared to a wait-list control group on stress in college students with elevated stress (≥14 on the Perceived Stress Scale [PSS]). We also explored the feasibility (ie, acceptability and demand) of the intervention delivered via the mobile app and examined the sustained effects (at 12 weeks from baseline) of the intervention on stress, mindfulness, and self-compassion. Additionally, exploratory analyses examined the potential effects of the mindfulness intervention on health behaviors (ie, sleep disturbance, alcohol consumption [binge drinking], physical activity, and healthy eating [fruit and vegetable consumption]).

We hypothesized that college students in the intervention group would have significant improvements in perceived stress as compared to the wait-list control. We also hypothesized that stress, mindfulness, and self-compassion would have sustained effects in the intervention group as compared to the wait-list control. Finally, we hypothesized that the intervention may improve health behaviors. The findings from this study will provide important insights on the potential stress-reducing effects of a consumer-based mindfulness meditation mobile app (ie, Calm) and could be applied to the design of future mindfulness meditation interventions in stressed college students.

## Methods

### Ethics Approval

This study was approved by an Institutional Review Board at a large university in the Southwestern United States (STUDY00006896). All participants provided electronic consent prior to participation in the study. The datasets generated or analyzed during the study are available from the corresponding author upon request.

### Study Design

This study was a randomized, wait-list control trial (trial registration: ClinicalTrials.gov NCT03891810) with assessments conducted at baseline, postintervention (8 weeks), and at follow-up (12 weeks). Participants randomized to the intervention group participated in an 8-week mindfulness meditation mobile app intervention of at least 10 minutes per day. Those randomized to the wait-list control group received the intervention after 12 weeks.

### Sample Size

The sample size was determined to detect significant change in perceived stress between the intervention and control groups (effect size=0.76) based on previous studies [28,54]. The power analysis showed that 93 participants were sufficient to have 85% statistical power at a two-sided α of 0.05 for significance. Accounting for a 10% dropout rate, the total sample size was planned to be 104 participants.

### Recruitment and Selection

Participants were recruited between January and April 2018 via social media (ie, Facebook and Instagram), email listservs, and flyers and by emailing university professors. Interested students were sent an eligibility survey via Qualtrics (ie, online survey database). Participants were eligible for the study if they were a current full-time undergraduate student attending a university in the Southwestern United States and (1) at least 18 years old, (2) scored ≥14 points on the PSS, (4) owned a smartphone, (5) were willing to download the Calm app, (6) were willing to be randomized, and (7) were able to read and understand English. Participants were excluded if they had a current mindfulness practice (ie, practice ≥15 min per day of meditation, yoga, and body scan within the past 6 months) [55] or were currently using the Calm app or another meditation app. Eligible participants were sent a link via Qualtrics to an online intake video that was approximately 5 minutes long. Participants were required to view the video in its entirety and correctly answer three follow-up questions regarding information presented in the video (eg, how long will you be asked to participate in mediation?). If participants missed a question, they were redirected to watch the video again and answer the questions until all three were answered correctly. Once the questions were answered correctly, participants were sent the informed consent and baseline questionnaire via Qualtrics software (Qualtrics, Provo, UT). After the informed consent and baseline questionnaire was completed, participants were randomized into the intervention group or the wait-list control group.

### Randomization and Blinding

Participants were randomized using a list created from an online randomization tool (randomizer.org) that was set to randomize in a ratio of 1:1. After participants completed baseline assessments, a research team member (unblinded) allocated participants to a group using the randomization list. Study staff and participants were unblinded to group allocation.

### Intervention Group

After randomization, the intervention group was emailed instructions on how to download the Calm mobile app. Calm is a consumer-based mindfulness meditation mobile app that offers a range of mindfulness meditation practice guide modules that vary in length, instruction, and content. Mindfulness meditation is the practice of moment-to-moment awareness in which the person purposefully focuses on the present without judgement. Vipassana is a technique of mindfulness that explores how the mind influences the body and how the body influences the mind (ie, objective observation of physical sensations in the body) [56]. The goal is to sharpen concentration, maintain awareness, and develop equanimity by releasing habitual tendencies toward craving and aversion. Calm also integrates some cognitive behavioral therapy (CBT) techniques into the meditation sessions on occasion. The CBT-influenced sessions encourage users to develop awareness of their thoughts, interpretations, and emotional and physiological responses in order to alter their perception of a situation or create a new, more balanced thought process [57].

Participants can meditate using the “daily Calm” set of guided meditations or may choose from a number of programs offering multiday meditations specific to goals (ie, happiness or self-esteem). Calm also offers other individual guided and unguided (eg, a brief introductory guidance followed by a chosen period of silence or sounds from nature) meditations.

Once downloaded, participants were asked to complete the “7 Days of Calm” program for the first week of the intervention to familiarize themselves with the principles of meditation and to standardize the introductory teaching content. Each day, the “7 Days of Calm” program started with an educational component on a principle of mindfulness meditation (eg, being present, returning to the here and now, and pulling out of autopilot). For each 10-minute session, after a principle was discussed, a related mindfulness meditation exercise was introduced and guided (eg, body scan, breath focus, and loving kindness). After participants completed the “7 Days of Calm” program, they were allowed to choose any meditation they desired. They could choose meditations from the College Collection, which addresses topics such as stress, sleep, self-compassion, and concentration or they could choose another series such as “7 Days of Managing Stress.” During the 8-week intervention, participants were asked to complete at least 10-minutes daily of meditation and could exceed that time limit by choosing additional meditations. If participants were not achieving 30 minutes of meditation per week, they were sent a text reminder to meditate. After the 8-week intervention, participants still had access to calm and could use it at their own leisure for 1 additional month (12 weeks from baseline).

### Wait-List Control Group

Participants randomized to the wait-list control group received an email with their group assignment and stating that they would receive access to the Calm app after 12 weeks. They were also asked not to participate in any mindfulness activities (eg, yoga, meditation, and qigong) during this time. After 8 weeks, participants received a Qualtrics link to the postassessment (same surveys that the intervention group received). After 12 weeks, participants were sent a Qualtrics link to the follow-up assessment (same surveys that the intervention group received) and an email with instructions on how to download Calm and the assigned username and password.

### Measures and Incentives

Both the intervention and wait-list control groups were administered three surveys—at baseline (week 0), postintervention (week 8), and at follow-up (week 12)—to assess perceived stress, mindfulness, self-compassion, health behaviors, and feasibility outcomes via online surveys (Qualtrics). Data on demographics, mental health history, medication use, and counseling activities were collected at baseline. Participants were incentivized with a US $5 gift card for completing baseline questionnaires, US $10 gift card for completing the postintervention questionnaires, and US $15 gift card for completing follow-up questionnaires. Participants could choose to receive their gift card from Starbucks, Target, or Amazon. Calm memberships were provided to participants for free during the study.

#### Perceived Stress

Stress was measured using the PSS [58,59]. This scale is a 10-item inventory used for the assessment of perceived stress. The scale measures the degree to which situations are appraised as stressful. The response items are rated on a 5-point Likert scale ranging from 0 (never) to 4 (very often). Scores range from 0 to 40, with higher scores indicating higher levels of perceived stress. The PSS has been shown to be reliable in undergraduate college samples [58,59]; in this study, the alpha coefficient for baseline PSS scores was 0.82.

#### Mindfulness

Mindfulness was measured using the Five Factor Mindfulness Questionnaire (FFMQ) [60]. The FFMQ is a 39-item self-report inventory used for the assessment of multiple constructs of mindfulness skills. The inventory assesses five subscales: observing, describing, acting with awareness, nonjudgment of inner experience, and nonreactivity to inner experience. The response items are rated on a 5-point Likert scale ranging from 1 (never or very rarely true) to 5 (very often or always true). The facet scores range from 8 to 40, with the exception of nonreactivity to inner experience, which ranges from 7 to 35. Higher scores indicate higher levels of mindfulness. The five FFMQ subscales have high internal reliability, test-retest reliability, and validity in undergraduate samples [60]. Consistent with this research, the FFMQ subscale reliability in this study was found to be high (at baseline, alpha coefficients were between 0.80 and 0.92).

#### Self-Compassion

Self-compassion was measured using the Self-Compassion Survey Short-Form (SCS-SF) [61]. The SCS-SF is a 12-item survey assessing three subscales: self-kindness versus self-judgment, common humanity versus isolation, and mindfulness versus over-identification. The response items are rated on a 5-point Likert scale ranging from 1 (almost never) to 5 (almost always). Higher scores indicate higher levels of self-compassion. The SCS-SF is a valid and reliable measure to assess self-compassion in college students (α=0.87 in our sample), and previous research has demonstrated near-perfect correlations (*r*=0.98) with the long-form version [61].

#### Secondary Outcomes

Health behaviors measured included sleep disturbance, alcohol consumption (ie, binge drinking), physical activity, and fruit and vegetable consumption. Sleep disturbance (ie, sleep quality) was measured using the Patient-Reported Outcomes Measurement Information System (PROMIS) (α=0.97 in our sample) [62]. The Youth Risk Behavior Surveillance (YRBS) survey was used to measure binge drinking, physical activity participation, and healthy eating [63]. Specifically, items from the YRBS survey inquired about whether participants had consumed alcohol (ie, engaged in binge drinking; for women, consuming four consecutive alcoholic beverages in a 2-hour period; for men, five alcoholic beverages) at any point during the past 7 days, engaged in at least 150 minutes of physical activity during the past 7 days, and eaten five servings of fruits or vegetables on most of the past 7 days.

#### Feasibility

Feasibility measures included acceptability and demand. Acceptability was measured with a satisfaction survey postintervention (week 8). Demand was measured using adherence (minutes/week) to the meditation intervention. Adherence to meditation was recorded in weekly reports from the Calm informatics team. Reports included the date and time of each meditation participated in, the title of the meditation, and the duration of participation (ie, the time spent viewing the meditation) for each participant.

### Statistical Analyses

General linear models (GLMs) were used to examine differences in means between groups at baseline and to test the initial efficacy of the intervention, examining mean differences of change (ie, change scores) for perceived-stress, mindfulness, and self-compassion after 8 weeks of mindfulness meditation between the intervention and control groups after adjustment for covariates (age, gender, and race). To examine the sustained effects, linear mixed models were used to test mean differences for perceived stress, mindfulness, and self-compassion between groups, time, and group×time interaction factors, with adjustment for covariates (age, gender, and race) performed using Bonferroni posthoc tests. GLMs were also used to evaluate changes in sleep disturbances; however, because sleep was exploratory, covariates were not included in these models. The McNemar tests were used to examine changes in other exploratory outcome variables (ie, binge drinking, physical activity, and healthy eating), which were coded dichotomously.

Feasibility potential was examined using adherence to the intervention. Adherence was calculated by averaging the weekly meditation minutes provided by the reports from the Calm informatics team. Acceptability measures and type of meditation were summarized using frequency with percentages. The Cohen *d* statistic was used to compute effect size. A two-sided α of 0.05 was used to determine statistical significance between groups, and a Bonferroni correction α of 0.016 was used for within-group comparison. All statistical procedures were performed using the IBM Statistical Package for Social Sciences (SPSS) version 24.0 (IBM Corp, Armonk, NY) and Statistical Analysis Systems Software, version 9.4 (SAS Institute, Cary, NC).

## Results

### Participant Enrollment

**Figure 1:**
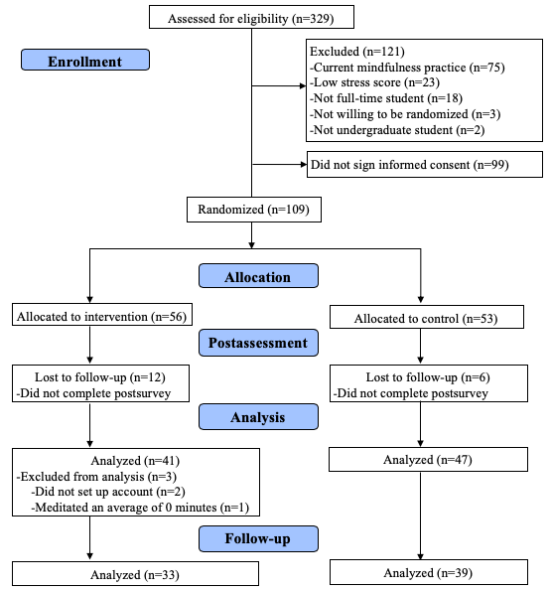
Enrollment flow diagram.

A total of 329 Arizona State University students completed the eligibility questionnaire. Of those who met the study eligibility, 37% (n=121) were ineligible and 30% (n=99) did not sign the informed consent. Subsequently, 56 participants were randomized to the intervention group and 53, to the wait-list group, yielding a total of 109 consented/randomized participants. We excluded participants who did not complete the postsurvey (n=18), set up a Calm account (n=2), or meditate at all (n=1), resulting in a total of 88 participants to be included in the analysis (Figure 1).

### Participant Demographics

Baseline characteristics of the study participants are presented in Table 1. The mean age (SD), adjusted for gender and race, was 20.41 (2.31) years for the intervention group and 21.85 (6.3) years for the control group. The majority of participants were female (79/88, 88%), freshman (27/88, 31%), non-Hispanic (66/88, 77%), and of white race (48/88, 59%). There were no differences in demographic characteristics, mental diagnosis, medication use, and counseling activities between the intervention and control groups (using Chi-square tests, all *P*>.17).

**Table 1:** Baseline characteristics of the study participants (N=88).

Characteristics		Intervention (n=41)		Control (n=47)	*P* value
Age (years)^a^, mean		20.41		21.85	.18
**Gender, n (%)**					.57
	Male		5 (6)	4 (5)	
	Female		36 (41)	43 (49)	
**Class, n (%)**					.61
	Freshman		10 (11)	17 (19)	
	Sophomore		12 (14)	10 (11)	
	Junior		10 (11)	12 (14)	
	Senior		9 (10)	8 (9)	
**Ethnicity, n (%)**					.17
	Hispanic		13 (15)	7 (8)	
	Non-Hispanic		27 (31)	39 (44)	
	Prefer not to respond		1 (1)	1 (1)	
**Race, n (%)**					.54
	White/Caucasian		25 (28)	23 (26)	
	Asian/Asian American		6 (7)	9 (10)	
	Black		1 (1)	4 (5)	
	Biracial/multiracial		3 (3)	7 (8)	
	Other		3 (3)	2 (2)	
	Prefer not to respond		3 (3)	2 (2)	
**Mental diagnosis, n (%)**					.53
	Yes		12 (14)	11 (13)	
	No		29 (33)	36 (41)	
**Medications, n (%)**					.94
	Yes		5 (6)	6 (7)	
	No		36 (41)	41 (47)	
**Counseling, n (%)**					.20
	Yes		2 (2)	6 (7)	
	No		39 (44)	41 (47)	

^a^Adjusted for gender and race.

### Primary Outcomes (Perceived Stress, Mindfulness, and Self-Compassion)

Table 2 describes the means of perceived stress, mindfulness, and self-compassion between the intervention and control groups at baseline, postintervention, and at follow-up. The baseline mean (SD) scores of self-reported stress (intervention: 23.11 [4.93] vs control: 21.88 [4.94]; *P*=.25), mindfulness (intervention: 109.41 [17.36] vs control: 113.88 [17.35]; *P*=.25), and self-compassion (intervention: 29.81 [8.13] vs control: 31.85 [8.2]; *P*=.25) showed no significant differences between study groups.

Table 3 summarizes the results of 8 weeks of meditation on changes in stress, mindfulness, and self-compassion, testing the initial efficacy of the intervention compared to wait-list control participants. Participants in the intervention group had a significant reduction in perceived stress compared with the control group (∇=–7.13; *P*<.001; effect size=1.24). Participants in the intervention group had significant improvements in total mindfulness (∆=19.23; *P*<.001; effect size=1.11) and all five constructs: observe (∆=3.735; *P*<.001; effect size=0.67), describe (∆=3.528; *P*<.001, effect size=0.59), acting with awareness (∆=4.737; *P*<.001; effect size=0.83), nonjudgment of inner experience (∆=4.938; *P*<.001; effect size=0.76), nonreactivity to inner experience (∆=3.781; *P*<.001; effect size=0.92). Participants in the intervention group also had significant improvements in self-compassion (∆=8.223; *P*<.0001; effect size=0.84) compared to participants in the control group.

**Table 2:** Mean scores per study group for perceived stress, mindfulness, and self-compassion.

Variable		Baseline (n=88)						Postintervention (n=88)						Follow-up (n=71)				
		Intervention			Control			Intervention			Control			Intervention			Control	
		n	Mean (SD)	n		Mean (SD)	n		Mean (SD)	n		Mean (SD)	n		Mean (SD)	n		Mean (SD)
												
PSS^a^		41	23.11 (4.93)	47		21.88 (4.94)	41		16.15 (6.16)	47		20.02 (6.16)^b^	32		15.89 (6.71)	39		19.86 (6.70)^b^
**FFMQ^c^**		38	109.41 (17.36)	45		113.88 (17.35)	40		129.20 (18.32)	44		111.07 (18.31)^d^	32		132.50 (20.83)	39		114.82 (20.81)^d^
	Observe	40	23.96 (5.83)	47		24.40 (5.84)	41		27.80 (5.60)	47		23.17 (5.60)^d^	32		28.07 (5.75)	39		24.94 (5.74)^e^
	Describe	40	23.22 (6.08)	46		23.75 (6.08)	41		26.75 (5.85)	46		23.48 (5.90)^b^	32		28.11 (7.68)	39		24.03 (6.88)^b^
	Act aware	39	21.62 (6.13)	47		21.80 (6.10)	40		26.42 (5.38)	46		21.64 (5.41)^d^	32		26.77 (5.66)	39		22.22 (5.71)^b^
	Nonjudgment	39	20.95 (6.75)	46		24.02 (6.79)^e^	41		25.88 (6.21)	46		23.04 (6.28)^e^	32		27.29 (6.72)	39		23.15 (6.71)^b^
	Nonreactivity	40	18.89 (4.39)	47		19.65 (4.39)	41		22.72 (3.97)	47		19.76 (3.95)^d^	32		22.25 (4.12)	39		20.49 (4.12)
SCS-SF^f^		39	29.81 (8.13)	46		31.85 (8.20)	40		37.06 (9.02)	47		31.91 (8.92)^d^	32		39.16 (8.97)	39		33.66 (8.96)^b^

^a^PSS: Perceived Stress Scale.

^b^*P*<.01.

^c^FFMQ: Five Facet Mindfulness Questionnaire.

^d^*P*<.0001

^e^*P*<.05.

^f^SCS-SF: Self-Compassion Scale - Short Form.

**Table 3:** Pre-post change scores in perceived stress, mindfulness, and self-compassion between groups (N=88; all *P*<.001). Scores are adjusted for age, gender, and race.

Variable		Change score	
		Intervention (n=41)	Control (n=47)
PSS^a^		–7.137	–2.013
**FFMQ^b^**		19.23	–2.408
	Observe	3.735	–1.272
	Describe	3.528	–0.257
	Act aware	4.737	–0.260
	Nonjudgment	4.938	–0.838
	Nonreactivity	3.781	0.138
SCS-SF^c^		8.223	0.294

^a^PSS: Perceived Stress Scale.

^b^FFMQ: Five Facet Mindfulness Questionnaire.

^c^SCS-SF: Self-Compassion Scale - Short Form

**Figure 2:**
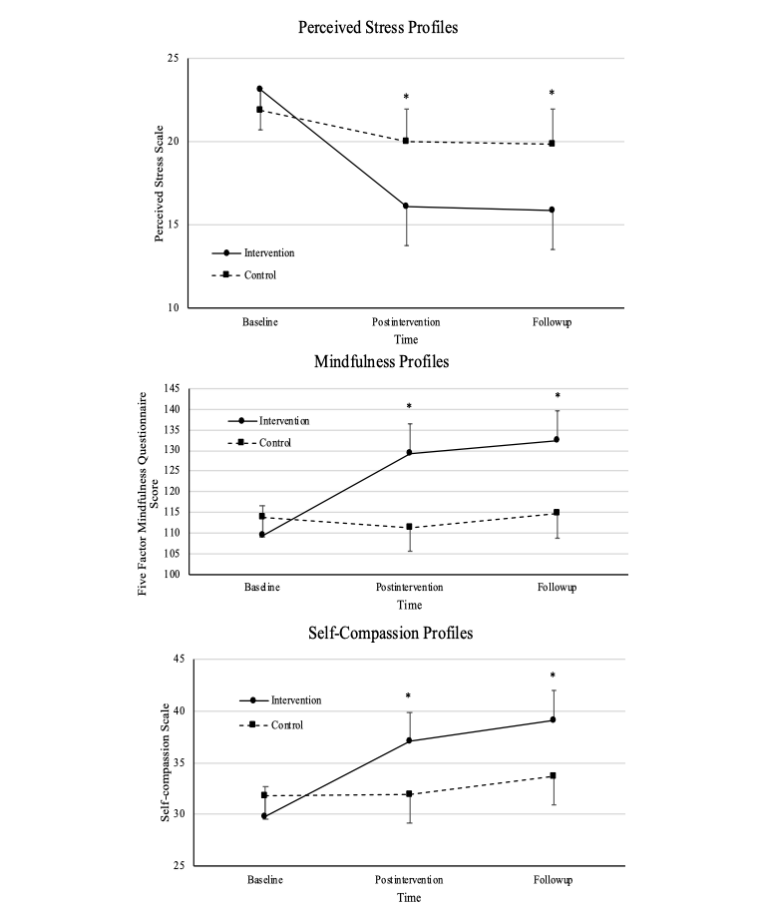
Effects of mindfulness meditation on perceived stress, mindfulness, and self-compassion.

Additional analyses using linear mixed models were used to examine effects of the intervention compared to control over time, including initial and sustained effects. Figure 2 illustrates the means of perceived stress, mindfulness, and self-compassion and the 95% CIs between intervention and control groups across baseline, postintervention, and at follow-up. On an average, participants in the intervention group had significantly decreased perceived stress scores postintervention (8 weeks) as compared to those in the control group, and these changes persisted through follow-up (12 weeks; Figure 2 and Table 2). Compared to those in the control group, participants in the intervention group showed significantly increased mindfulness and self-compassion scores postintervention (8 weeks), and these changes persisted through follow-up (12 weeks).

There was a significant interaction between group and time factors in perceived stress (*P*=.002), mindfulness (*P*<.001), and self-compassion (*P*<.001). Bonferroni posthoc tests showed significant within-group mean differences for perceived stress in the intervention group (baseline and postintervention: *P*<.001; baseline and follow-up: *P*<.001), while there were no significant within-group mean differences for perceived stress in the control group (all *P*>.19). For mindfulness and self-compassion, there were significant within-group mean differences in the intervention group (baseline and postintervention: *P*<.001; baseline and follow-up: *P*<.001), while there were no significant within-group mean differences in the control group (all *P*>.40). In the intervention group, there were no statistical differences in perceived stress, mindfulness, and self-compassion between the postintervention phase and follow-up (all *P*>.05).

### Secondary Outcomes (Sleep Quality, Binge Drinking, Physical Activity Participation, and Healthy Eating)

Linear models indicated that among participants using the mindfulness meditation app, there was a significant decrease in sleep disturbance, as indicated by *t* scores on PROMIS (*P*=.02; effect size=0.79), while there were no changes among control participants (*P*=.95; effect size=0.02). However, results from linear mixed models showed that the interaction between the time and intervention groups was nonsignificant, suggesting that sleep improvements did not differ across the two groups (*P*=.11; effect size=0.34).

Data from the three YRBS items were used to create dichotomous variables reflecting whether in the past 7 days, participants engaged in binge drinking (ie, consumed four or more [for women] or five or more [for men] alcoholic beverages in a row on at least one day), engaged in 150 minutes of physical activity, and ate five serving of fruits and vegetables per day on most days. Results from the McNemar tests indicate that there were no significant changes in binge drinking for students in either study group (all *P*>.51) for physical activity or eating fruits and vegetables.

### Acceptability (Satisfaction, Enjoyment, Intent to Continue Use)

We assessed acceptability via a satisfaction survey postintervention (week 8) in the intervention group. Approximately, 51% (21/41) of the students indicated that Calm was helpful to very helpful in reducing their stress in the short term, and these findings were similar for the long term (20/41, 49%). Over half of the students said they would use Calm to help reduce stress in the future. Approximately 85% (35/41) of the sample was somewhat to very satisfied using Calm, and 85% (35/41) indicated that they somewhat to very much enjoyed using Calm. About 68% (28/41) of the sample said they were likely to extremely likely to use Calm in the future, and 76% (31/41) would be likely to extremely likely to recommend Calm to other college students.

### Demand (Adherence)

Participants in the intervention group engaged in an average of 37.9 (SD 30.5) minutes of meditation per week over the course of the 8-week study. Over half of the participants (23/41, 56%) completed more than 30 minutes per week using the Calm app, with 22% (9/41) of participants meditating more than 60 minutes a week. Approximately one-third (14/41, 34%) of intervention participants continued to meditate during the follow-up period (12 weeks from baseline) and spent an average of 20.4 (SD 23.9) minutes meditating.

## Discussion

### Principal Findings

The purpose of this study was to test the efficacy and sustained effects of an 8-week mindfulness meditation intervention delivered using a consumer-based mobile app (ie, Calm) to reduce stress in college students and to examine feasibility components germane to planning the next steps in research. This was one of the first studies to test the efficacy of such a product in undergraduate college students. Our findings demonstrate significant between-group differences on all main outcomes variables including perceived stress, all five factors of mindfulness (observing, describing, acting with awareness, nonjudgment of inner experience, and nonreactivity to inner experience), and self-compassion postintervention. These effects were sustained at follow-up, and effect sizes ranged from moderate (0.59) to large (1.24) across all outcomes. Additionally, Calm was perceived as helpful; many participants enjoyed Calm and were likely to continue using the app. These findings highlight the promise of Calm to reduce stress in college students.

### Perceived Stress

To our knowledge, this study was one of the first to demonstrate the stress-reducing effects of a mindfulness meditation mobile app (ie, Calm) in college students. The magnitude of change in perceived stress was greater in the intervention group than in the control group postintervention and at follow-up. Our findings are similar to those of a recent study that tested whether two mindfulness meditation apps (Headspace and Smiling Mind) led to improvements in mental health as compared to a control group in undergraduate students (N=208) [64]. Students were asked to meditate for 10 minutes daily for 40 days (10 days of requested use followed by 30 days of discretionary use). Students using Headspace (not Smiling Mind) reported significant reductions in stress and were maintained at follow-up. However, unlike our study, the app usage was measured by self-report, nearly half of all participants never used the app during the open-access period, students did not have elevated levels of stress as part of eligibility criteria, and the effect size was small (*d*=0.26). Another study testing 10-20 minutes/day of Headspace use for 30 days among medical students (N=88) reported similar findings on stress compared to a wait-list control group both postintervention (30 days) and at follow-up (60 days) [65]. App usage in this study was self-reported, and participants were also instructed to send a screenshot to the research team of the minutes meditated report in Headspace. However, this study only reported on the days of app usage rather than minutes meditated and did not report effect sizes. Reporting days of app usage does not reflect the total amount of minutes meditated and does not illustrate how minutes of meditation may vary across days. Our findings are also similar to those of a study testing a newly developed mindfulness app (Wildflowers) on stress compared to a cognitive training control group (app called 2048) in college students (N=86) [66]. Students were asked to use the Wildflowers app daily for 3 weeks and could choose lessons on meditation or choose from a library of guided meditations. Students using the Wildflowers app had improved mood and reduced stress following each training session. However, the study did not have adequate power, did not report on the number of minutes students had meditated (only sessions completed), and had small effect sizes (*r*=–0.08 to –0.37). Although all the aforementioned studies demonstrated a reduction in the number of minutes (or sessions) meditated, ours was the longest intervention (8 weeks) and showed the strongest effect sizes for perceived stress (effect size=1.24).

Although the average minutes of meditation in our study significantly decreased at follow-up, impressively, the reduction in perceived stress remained significant at follow-up (12 weeks from baseline). Similar to findings of other studies, our findings suggest that brief mindfulness meditation interventions delivered via mobile apps show promise in reducing stress in college students, and there *may be* added benefit for weeks following the intervention period, regardless of the time spent in meditation. Delivering mindfulness meditation may be a more practical approach to reducing stress, as it requires fewer resources (eg, cost, staff, or brick and mortar building), students can participate remotely, and there are fewer time constraints. However, there is a need to independently test and determine the efficacy of mindfulness meditation using mobile apps.

### Mindfulness

We also demonstrated significant improvements with large effects (effect size=1.11) in overall mindfulness scores and significant improvements with moderate-to-large effects (effect size=0.59-0.92) in all five factors of the FFMQ (observing, describing, acting with awareness, nonjudgment of inner experience, and nonreactivity to inner experience). Yamada and colleagues [67] implemented a brief 10-minute mindfulness meditation intervention (led by an instructor twice a week) over a 15-week semester in college students. Those participating in the mindfulness group, on an average, increased their mindfulness scores by nearly 9% as compared to the control group (no change). However, investigators did not provide the mindfulness factor scores. Greeson and colleagues [42] implemented a mindfulness meditation intervention (Koru) in college students and found significant increases in mindfulness and large effect sizes at the end of the 4-week intervention compared to a wait-list control group [42]. The Koru program includes 75-minute sessions taught in a small group format and also requires 10-minute daily meditation practice. Both the aforementioned studies, and our current study, demonstrate that brief mindfulness meditation interventions have the potential to improve mindfulness scores; however, the aforementioned studies did not include a follow-up period to assess the sustained effects of the intervention. In our study, mindfulness was greater even at the follow-up assessment. More studies are needed to test the sustained effects of brief mindfulness meditation interventions.

### Self-Compassion

We found significant improvements in self-compassion in the intervention group as compared to the wait-list control group after the 8-week intervention with a large effect size (effect size=0.84). Improvements in self-compassion were sustained at follow-up. Greeson and colleagues (2014) also found significant increases with a large effect size in self-compassion, measured by the 26-item Self-Compassion Scale [68], as compared to a wait-list control group [42], but did not include a follow-up assessment. Another study implemented a brief mindfulness intervention (based on MBSR) that included 10-minute periods of mindfulness training followed by 5 minutes of discussion across 28 clinical interviewing classes (7 hours of instruction over 10 weeks) in social work students [69]. In contrast to our findings, this study did not find significant improvements in self-compassion. However, this study was limited by a lack of randomization and a small sample size. Despite these findings, our study and others demonstrate that self-compassion often improves with increasing mindfulness in students [29,36,70], but the sustained effects after the intervention period are unclear. Self-compassion has been considered a core quality of mindfulness [71] and is associated with lower stress and psychological symptoms in addition to greater well-being [72,73]. In one study, mindfulness and self-compassion were mediators in the pathway to emotional well-being in adolescents [36]. More research investigating the impact of brief mindfulness meditation interventions, especially those delivered via mobile apps, on self-compassion is needed to determine the efficacy of such apps and potential to sustain effects after the intervention period.

### Sleep Quality and Health Behaviors

Our findings suggest that Calm may be a promising approach to help improve sleep quality (ie, reduce sleep disturbance) in college students. Similar to our findings, Greeson and colleagues [42] reported a significant reduction in sleep problems as compared to a wait-list control group after participation in a 4-week mindfulness meditation intervention. However, the intervention was only for 4 weeks and delivered in person. Another study tested the impact of an 8-week internet-based mindfulness training program to improve mental health and sleep in college students and young adults compared to an internet-based cognitive behavioral training program [74]. Each session lasted 30-45 minutes and both groups experienced significant reductions in sleep disturbance. This study also did not deliver the intervention with a mobile app and had a high attrition rate. One study tested a mindfulness-based health app on mental health and sleep behavior in college students [75]. Students were asked to use the app 5 days per week for 4 weeks, but there were no significant changes in sleep. There are limited and mixed data available on the impact of mobile meditation apps and sleep outcomes in college students, and there is a need for further exploration of meditation delivered via an app and sleep outcomes in college students.

We did not find an association between mindfulness meditation and changes in alcohol consumption (ie, binge drinking), physical activity, or healthy eating (ie, fruit and vegetable consumption). There are a lack of studies testing the impact of mindfulness meditation mobile apps on health behavior outcomes in college students. One study testing the effects of a mindfulness app on weight and weight-related behaviors reported reductions in emotional eating and uncontrolled eating after engagement in an 11-week intervention compared to a self-monitoring electronic diary control group [76]. However, this study had low adherence and did not report on fruit and vegetable consumption. A cross-sectional survey administered to college women reported that higher levels of mindfulness were related to healthy eating practices, better sleep quality, and better physical health [77]. Another cross-sectional survey implemented in adults aged 18-25 years suggested that self-compassion (a potential mechanism of mindfulness) was positively correlated with intentions to engage in health-promoting behaviors (eg, healthy eating, physical activity, good sleep hygiene, and stress management) [78]. Papies (2017) suggests that mindfulness interventions may be most effective if targeted at a specific behavior (ie, reducing alcohol consumption, healthy eating, and reducing smoking) [79]. Although mindfulness-based interventions show promise in improving health behaviors, more research in this area is warranted.

### Feasibility

Approximately 85% of the sample was satisfied with and found Calm enjoyable. None of the students in our sample indicated that they were dissatisfied with using Calm. A majority said they would use the app in the future or refer it to other college students. There is limited evidence regarding the acceptability of mindfulness meditation mobile apps, specifically in college students. Similar to our data, a study by Donovan and colleagues [80] suggested high satisfaction with a mobile app (BodiMojo) aiming to teach adolescents (13-22 years old) about mindfulness and self-compassion, with 92% of the sample rating their enjoyment of the program a score of 6 or 7 (1=not at all, 7=very much) [80]. Another study conducted in the Netherlands by Emmerik and colleagues [81] implemented a mindfulness-based mobile app (VGZ Mindfulness Coach) to improve mindfulness, general psychiatric symptoms, and quality of life in adults aged ≥18 years [81]. Participants in the study reported high satisfaction with an average satisfaction score of 4.18 (SD 0.84) on a scale of 1-5 (higher scores indicate higher satisfaction) [81]. The aforementioned research is similar to our findings and demonstrates high satisfaction among mindfulness-based mobile apps. However, it is difficult to compare the mobile apps against each other because of the various mindfulness components used, differences in application interfaces, usability, and length of programs. Future studies may assess the satisfaction of the various components of behavioral/mental health mobile apps, as studies have reported that satisfaction of these apps may be dependent upon components such as accountability, tracking progress, reminders/push notifications, and convenience [82,83]. This information could be particularly useful for researchers to design/modify new or existing mindfulness meditation mobile apps to be more relatable to the user and potentially attenuate the decline in usage over time.

The average participation in meditation across the 8-week study was 38 minutes/week, which was sufficient to see effects in perceived stress in our sample. The decrease in minutes of meditation practiced by college students over the length of the intervention is not surprising, as college students have a number of barriers that may keep them from participating in daily meditation (eg, time constraints, financial pressure, educational expectations, and interpersonal relationships). In a formative analysis by Lauricella [84], a majority of undergraduate students who participated in a face-to-face mindfulness meditation intervention followed by a digital session of the same technique preferred the in-person version as compared to the digital practice [84]. The minority (~25%) that preferred the digital practice found the solitude and confidentiality of this format to be favorable. It is important to understand more about why college students do or do not participate in meditation delivered digitally. Gender differences regarding use of mindfulness-based mobile apps may be important considerations in future studies. Men are often underrepresented in mindfulness interventions, and women may be more engaged in the intervention [85] and experience greater benefit [86]. We also provided students with incentives, which may have impacted intervention results and adherence. Future studies may consider replicating this study without the use of incentives to observe real-world participation. Furthermore, many consumer-based mobile apps do not include theory-based strategies for behavior change, which may impact adherence rates [87]. For example, self-monitoring (ie, the ability to track minutes) and social support are strategies highly associated with adherence to behavior. Calm is a consumer-based app that does include tracking and reminders for participants, but other strategies may be necessary. Future studies could incorporate social support with an app and examine the effects of such an addition on adherence.

It is also important to note that mobile app usage rapidly declines within the first 90 days of downloading the app [88]. According to a 2018 report by Localytics, 21% of users abandon using an app after only one use, 57% of users stop using the app within the first 4 weeks, and average retention by 90 days is only 29% across all industries (media and entertainment, electronic commerce/retail, travel and lifestyle, business, and technology) [88,89]. Several studies that have investigated barriers to health-related mobile app usage have identified a lack of regulatory oversight, limited evidence-based literature, privacy/security concerns [87], poor functionality or incompatibility, lack of desired features, abandonment of a health goal [90], loss of interest, hidden costs, and data entry burden [91]. Mobile apps have the potential to provide affordable, convenient access, and evidence-based information to a large and broad audience. However, as noted previously, there are several limitations that need to be addressed to improve the quality and efficacy of mobile apps.

### Limitations

Although the findings in this study are promising regarding the efficacy of a mindfulness-based mobile app to reduce stress and improve mindfulness and self-compassion among college students, with moderate-to-large effect sizes, several limitations must be noted. First, the sample mostly comprised white, young female adults; therefore, the generalizability of our findings may be limited. In future interventions, it is important to explore efficacy in more diverse samples (eg, race/ethnicity, education level, and chronic disease status) [44,54,92]. At the time of the intervention, Calm was only offered in English. Exploration of the efficacy of Calm with non-English speaking populations (and offered in other languages) is warranted. Second, all data collected in the study relied on self-report, which may lead to social desirability or response bias where participants respond in what they believe is a more favorable manner (although the wait-list control condition assists partially in controlling for this bias). We had a wait-list control group and were not able to control for maturation or expectation effects. Future studies warrant determining the best control condition. In our study, we tracked the minutes of meditation and not the frequency of sessions. Other studies have tracked both but only conducted analysis with one or the other [65,66]. Future studies should consider measuring and reporting both minutes and frequency (eg, days/sessions) related to participation. Our estimated sample size of 104 was not achieved (88 included in data analysis), but we did have adequate power because our effect sizes were strong. However, analyses of all secondary outcomes were exploratory and not sufficiently powered to detect an effect, and results for these outcomes should be interpreted with caution. Finally, we did not collect information regarding students’ school schedule, homework load, or testing schedule, and therefore, it is unclear how these factors may have played a role in students’ participation or stress scores.

### Conclusions

The findings in this study demonstrate the efficacy of Calm to reduce stress and improve mindfulness and self-compassion in short-term contexts in stressed college students. Although direct comparisons are not possible, it appears that the degree of improvement in response to a smartphone-based mindfulness app may be similar to programs that require in-person attendance with even greater opportunity for convenient compliance and continued use. Students also reported high satisfaction using Calm to reduce stress. Currently, there is limited literature exploring the effectiveness and acceptability of mobile apps for delivering mindfulness meditation. More research in this area is needed to establish efficacy and to explore the degree to which effects are sustained in the short and long term. Particularly, we suggest future independent replications of this work to establish efficacy. The findings presented here provide important information that can be applied to the design of future studies or mental health resources in university programs. Calm may be a cost-effective, convenient, easily disseminated, and enjoyable way to manage stress among college students.

## Appendix

Multimedia Appendix 1 CONSORT‐EHEALTH checklist (V 1.6.1).

## Abbreviations

CBT: cognitive behavioral therapy
FFMQ: Five-Factor Mindfulness Questionnaire
GLM: general linear model
MBCT: Mindfulness-Based Cognitive Therapy
MBSR: Mindfulness-Based Stress Reduction
PROMIS: Patient-Reported Outcomes Measurement Information System
PSS: Perceived Stress Scale
SCS-SF: Self-compassion Scale Short-Form
SPSS: Statistical Package for Social Sciences
YRBS: Youth Risk Behavior Surveillance
